# Neuroimaging Scoring Tools to Differentiate Inflammatory Central Nervous System Small-Vessel Vasculitis: A Need for Artificial Intelligence/Machine Learning?—A Scoping Review

**DOI:** 10.3390/tomography9050144

**Published:** 2023-10-02

**Authors:** Alameen Damer, Emaan Chaudry, Daniel Eftekhari, Susanne M. Benseler, Frozan Safi, Richard I. Aviv, Pascal N. Tyrrell

**Affiliations:** 1 Department of Medical Imaging, University of Toronto, Toronto, ON M5T 1W7, Canada; 2 Department of Radiology, Radiation Oncology and Medical Physics, University of Ottawa, Ottawa, ON K1N 6N5, Canada; 3 Department of Pediatrics, Cumming School of Medicine, University of Calgary, Calgary, AB T2N 4N1, Canada; 4 Institute of Medical Science, Department of Statistical Sciences, University of Toronto, Toronto, ON M5G 1X6, Canada

**Keywords:** artificial intelligence, machine learning, small-vessel vasculitis, CNS, inflammatory, neuroimaging

## Abstract

Neuroimaging has a key role in identifying small-vessel vasculitis from common diseases it mimics, such as multiple sclerosis. Oftentimes, a multitude of these conditions present similarly, and thus diagnosis is difficult. To date, there is no standardized method to differentiate between these diseases. This review identifies and presents existing scoring tools that could serve as a starting point for integrating artificial intelligence/machine learning (AI/ML) into the clinical decision-making process for these rare diseases. A scoping literature review of EMBASE and MEDLINE included 114 articles to evaluate what criteria exist to diagnose small-vessel vasculitis and common mimics. This paper presents the existing criteria of small-vessel vasculitis conditions and mimics them to guide the future integration of AI/ML algorithms to aid in diagnosing these conditions, which present similarly and non-specifically.

## 1. Introduction

Multiple sclerosis (MS) is a demyelinating central nervous system (CNS) disease. Previously, healthy individuals were typically diagnosed with MS in their 20s and 30s; females are affected two to three times more frequently [1,2]. In patients with MS, the body’s immune system attacks the myelin sheath of CNS neurons, resulting in damage to nerve fibers. Functional deficits, movement abnormalities, and cognitive deficits are commonly seen in patients with MS. It is a lifelong disease that currently has no cure; disease course and severity of neurological symptoms are variable [3]. Initial presenting symptoms are equally variable depending on the type of symptom experienced and the location of the lesion. Abnormal brain magnetic resonance imaging (MRI) is found in over 80% of clinically isolated syndrome patients who subsequently develop MS. MRI is considered the key diagnostic modality for a patient with suspected MS [4]. It is one of the common conditions that can mimic CNS vasculitis, yielding some difficulty in diagnosis.

Vasculitis of the CNS is characterized by inflammation primarily targeting the blood vessels of the brain and/or spinal cord, leading to potentially devastating injuries in previously healthy individuals [5]. CNS vasculitis can affect arterial vessels of all sizes in the brain or spine. A wide range of neurological symptoms, including headaches, stroke features, seizures, movement abnormalities, and cognitive deficits, are found in patients with CNS vasculitis. Diagnosis time and initiation of treatment are critical since inflammatory vessel wall changes can be reversible, and neurologic deficits can be significantly mitigated or avoided [6]. 

Large-vessel inflammation primarily results in reduced blood flow through the affected vessels, causing ischemia and cell death within the associated vascular territory. Patients present with stroke-like characteristics, including focal neurological deficits such as hemiplegia. Large-vessel disease can also be characterized by the involvement of the intracranial segment of the internal carotid artery, proximal anterior cerebral artery, middle cerebral artery, and/or the posterior cerebral artery. 

Small-vessel vasculitis (SVV) is defined as involving any vascular segments beyond the second-order branches [7]; inflammation is associated with significant involvement of the perivascular brain tissue. In SVV, there is a female predominance. Clinical features differ depending on age: 80% of children present with seizures, while adults show more diffuse neurological deficits such as communication problems, hallucinations, and depression; in older individuals, symptoms can be hard to differentiate from dementia. Elective brain biopsy is necessary and mandatory to diagnose SVV [8] definitively. This paper reviews the existing criteria to differentiate SVV from mimics. 

Neuroimaging, including MRI, MR angiography (MRA), and conventional angiography (CA), are key diagnostic modalities in CNS inflammatory disease. In suspected CNS vasculitis, the diagnosis is often suggested via MRI/MRA and/or CA findings but requires a brain biopsy for confirmation, as other conditions, such as infections, cancers, and MS, can mimic vasculitis [9]. Large-vessel disease is associated with specific MRI features and angiography characteristics, and MRI remains the gold standard for diagnosing medium and large-vessel vasculitis. MRA and/or CA may show cerebral vessel wall stenosis and MRI abnormalities in the associated vascular territory or can be completely normal in SVV. The affected vessels involved may be too small to be captured via angiography [10]. MRI lesions, however, are usually present in this group but can differ greatly among cases. MRI lesions are often non-specific and overlap with other inflammatory brain conditions, such as MS, and some CNS infections, such as Lyme disease. Consequently, a complete infectious work-up is recommended [11]. Emerging technology, including high-resolution vessel wall imaging and three-dimensional sequences, provides great promise in advancing our diagnostic ability through increased demonstration of inflammation and enhancement in vessel walls. Higher-resolution imaging allows for a greater ability to evaluate more distal, smaller vessels, which may be used to distinguish vasculitic from non-vasculitic etiologies better [10]. Vessel wall imaging also allows for direct characterization of vessel walls compared to conventional imaging, which relies on changes to the lumen [12]. 

While MRI and MRA are fast, non-invasive, and sensitive procedures to diagnose large vessel vasculitis, these investigations lack specificity and are less reliable in diagnosing SVV. To date, no established tool or method is available to differentiate between SVV and common mimics such as MS [13]. Establishing such a tool to aid in differentiation may be of clinical importance as treatment and diagnosis often rely heavily on radiological evaluation. Additional imaging techniques, such as these, will only enhance current scoring tools, providing additional context and detail to aid in the differentiation of SVV. AI/ML was a recurring theme in the papers we retrieved, indicating interest in its application within this field. For this reason, we have elected to discuss the role AI/ML may play and its promise in aiding the differentiation of CNS vasculitis conditions. The use of AI/ML may allow for the use of numerous imaging techniques and scoring tools at once.

This scoping review presents the scoring tools available within the research literature to diagnose various SVV conditions and mimics. The utility of an AI/ML solution based on these existing criteria/scores for augmenting our diagnosis and differentiation of these diseases is also explored.

## 2. Materials and Methods

### 2.1. Study Protocol and Data Acquisition

A scoping review of the literature was performed to assess the availability and utility of scoring tools to diagnose and differentiate between SVV and mimics. All original articles that describe criteria, scores, and/or neuroimaging findings (CA, MRI, and MRA) to evaluate small-vessel vasculitis diseases were included. The following clinical designs were included: retrospective, prospective, observational, and RCTs. Exclusion criteria included articles published prior to 2000, conference abstracts, languages other than English, reviews, studies that do not focus on the appearance of MRI, and studies that do not explain or focus on the diagnosis of the disease. 

### 2.2. Search Strategy

Searches were conducted in Ovid MEDLINE (1946–September 2023) and Ovid EMBASE (1974–September 2023). The search strategy was developed with the assistance of a trained librarian at our institution. These databases were selected in accordance with our institution’s protocol to create a search that would retrieve comprehensive and specific results. Numerous database-specific subject headings in each database (MeSH in MEDLINE, Emtree in EMBASE) were selected for both small-vessel vasculitis concepts, diagnostic imaging, and scoring/diagnosis tools. The following inflammatory brain diseases were included: demyelinating disease, acute disseminated encephalomyelitis, demyelination, leukodystrophy, multiple sclerosis, progressive multifocal leukoencephalopathy, Schilder disease, subacute sclerosing panencephalitis, central nervous system viral diseases, encephalitis, myelitis, autoimmune diseases of the nervous system, demyelinating autoimmune diseases of the central nervous system, demyelinating autoimmune diseases of the central nervous system, Rasmussen syndrome, stroke, primary angiitis of the central nervous system, and central nervous system vasculitis. Many of these subject headings were expanded, when applicable, to include narrower terms. If no relevant subject headings could be found for a specific disease, text word searches were used. All inflammatory brain disease terms were combined using the Boolean “OR” operator first. All imaging terms were combined using a Boolean “OR” operator. All diagnostic scoring tool terms were also combined using a Boolean “OR” operator. These three sets of terms were then combined with the Boolean “AND” operator. Results in both databases were limited to publications from 2000 to Sepember 2023. If scores were released prior to 2000 but were referenced in articles published from 2000 and onwards, they were included in our review. English language restrictions were applied. The full search string used is available in Appendix A.1.

### 2.3. Review Process

In accordance with the PRISMA guidelines for scoping reviews, two reviewers (EC and FS) independently screened all the titles and abstracts of the articles. The remaining articles were evaluated based on their full text to identify eligible studies. 

### 2.4. Quality Assessment

Selected articles were critically appraised independently by two reviewers based on methodological quality and content analysis using the STROBE guidelines to assess the quality of observational studies [14]. All articles were deemed satisfactory for inclusion. During this exercise, studies that were included were stratified on a scale of 1–4 based on their use of a score/criteria to diagnose vasculitis and/or mimics. 

Articles that were classified as type 1–4 were evaluated by the three investigators for the scoring tools or diagnostic criteria discussed, as well as items that described discriminatory neuroimaging features of inflammatory brain diseases.

- Type 1: The paper explicitly discusses or uses an established scoring tool to make their diagnosis;
- Type 2: The paper is improving a current scoring tool or diagnostic criteria;
- Type 3: The paper is working towards making an original scoring tool or establishing specific criteria for diagnosis;
- Type 4: These papers show that imaging was used for diagnosis; however, since they do not provide insight into how to identify vasculitis and/or mimics from imaging, it is not relevant to our research question.

## 3. Results

EMBASE yielded 947 articles, and Medline yielded 421 citations. A total of 1022 unique citations were identified after removing duplicates and excluding citations based on predefined criteria: language other than English and year of publication prior to 2000. After title and abstract screening, 563 were screened for full text. A total of 438 articles were excluded after screening the full text for the following reasons: 324 were conference abstracts, 67 were systematic literature reviews, 32 focused on the wrong patient population (e.g., non-vasculitis patients), 15 did not focus on neuroimaging, and 6 were the wrong study design (e.g., case reports). A total of 125 articles were classified as type 1–4, and data were extracted. Data included criteria(s) used, country of the institution, imaging discussed, main takeaways, pediatric vs. adult population, sample size, study type, and vasculitis condition studied. Data were independently extracted and reviewed by two authors and compiled into a spreadsheet (AD, EC). 

Of the 125 articles, 102 looked at an adult or mixed population, and 23 looked strictly at the pediatric population. Articles were classified into the following categories: 20 type one, 34 type two, 37 type three, and 34 type four papers. Of the type one papers, the most used score was the Calabrese and Mallek 1988 criteria or a rendition of it. A total of 13 of the 20 articles used this score or a modified version of the criteria. The second most commonly used scoring tools were the McDonald and Barkhof criteria. Other scores included the Bien et al. and Abu-Shakra et al. criteria. 

This pattern continues amongst the type two articles, of which 25 of the 34 papers used the Calabrese and Mallek criteria or a modified version. Other criteria used amongst these articles include the McDonald Criteria (including the multiple revised versions in 2005, 2010, and 2017), the Moore et al. criteria, the IPMS Criteria, Barkhof’s criteria, the Alrawi et al. criteria, Salvarani criteria, the MAGNIMS criteria, and the Wingerchuck Criteria. Of note, the 2017 revision of the McDonald’s criteria was included in the articles; however, it is not validated for children under 12. Recent studies have supported applying the 2010 McDonald’s criteria in children of any age when a clinically isolated syndrome (CIS) is explicitly documented [15,16]. 

One aim of our review was to investigate which neuroimaging scoring tools exist to evaluate CNS vasculitis diseases and mimics. As our results suggest, a wide range of tools exist in the literature to address this. Table 1 outlines the diagnostic criteria used in the literature for the vasculitis conditions, often on the differential. We hope that the presentation of these criteria can act as foundational knowledge for the future development of scores and criteria to diagnose and distinguish these conditions and as foundational data for implementing AI/ML to develop patterns to augment the ability to diagnose these conditions. 

## 4. Discussion

The diagnosis of CNS vasculitis has proved to be difficult given its ambiguous presentation, which overlaps with many other disease processes. MRI is the foremost imaging modality used in the work-up of suspected vasculitis. Sensitivity is high, ranging from 90 to 100% [22,25,36]. Findings on MRI are common as abnormalities have been reported in 97 and 98 percent of biopsy-confirmed and angiogram-confirmed cases, respectively [37]. Salvarani and colleagues report a 97% sensitivity and found that infarctions are the most common MRI findings, seen in 48 of 90 patients with primary angiitis of the CNS (PACNS) [22]. MRI abnormalities associated with vasculitis include changes in subcortical white matter, deep grey matter, deep white matter, and the cerebral cortex. Multiple infarcts can occur, as well as non-specific white matter lesions identified with T2-fluid-attenuated inversion-recovery sequence. Findings of hyperintense foci on T2 imaging can pose diagnostic problems given their low specificity, and thus, other causes should be ruled out, such as widened perivascular spaces, brain aging, migraines, ischemic changes, inflammatory changes, multiple sclerosis, metastases, radiotherapy and chemotherapy, lesions associated with neurometabolic diseases, and eclampsia [38].

Ultimately, differentiating these diseases proves difficult due to the many mimics [39]. The non-specific presentation, combined with the rarity of the condition, creates formidable difficulty in establishing the diagnosis [38]. 

### 4.1. Mallek and Calabrese

By far, the most commonly used score in the literature is the Mallek and Calabrese criteria [21]. This was proposed in 1998 and has since become the dominant diagnostic criteria for PACNS, as described in the Appendix A. This score has been used widely for diagnoses and the basis for research and the development of other scores and modified renditions of the score [38]. For example, the score expanded to include acquired neurological or psychiatric deficits to develop the criteria used for the pediatric score [26]. 

Calabrese and colleagues have since tested these criteria in 108 patients and derived the most common clinical features of the disorder [36]. Such features corroborated prior reviews, finding that the most common symptom is headache (62%). Other common symptoms include paresis (55.6%) and decreased cognition (50.9%). These were also the three most common symptoms reported by Salvarani et al.’s cohort of 101 vasculitis patients [22]. 

### 4.2. Other Diagnostic Modalities

Several other tools have been used to aid in diagnosis, including CSF analysis, electroencephalography, and high-resolution MRI (3 Tesla and above). CSF analysis, although invasive, is vital in excluding malignant or infectious disease processes or even demyelination. The combination of normal MRI findings and a normal CSF analysis denotes a high negative predictive value [40]. The drawback to CSF analysis is the invasive nature of lumbar punctures. Electroencephalography has also been shown to be a relatively sensitive marker, detecting abnormalities in 74% of patients [22]; however, the lack of diagnostic specificity limits electroencephalography as a standalone test [41]. High-resolution MRI (3 Tesla or greater) allows for greater discrimination of vessel wall imaging and may allow for better visualization. Currently, however, it is only capable of differentiating medium-sized vessel disease-specific vasculitis. With future implementation and development, hopefully, it will be of utility to image small vessels that are currently hard to visualize or unable to be seen on MRI [42]. Studies evaluating the diagnostic efficacy of high-resolution vessel wall imaging (HRVWI) on patients with PACNS have been promising. Sundaram et al. found that HRVWI was able to detect distinctive vessel wall appearances, specifically concentric vessel wall thickening and enhancement, yielding abnormalities in 95.2% of the patients in the study. Consequently, this suggested that HRVWI may be considered in the future as a non-invasive diagnostic modality for PACNS and possibly other SVDs [43].

### 4.3. Reversible Cerebral Vasoconstriction Syndromes (RCVS)

RCVS is one of the common mimics of vasculitis, including PACNS. It is rare but has become increasingly recognized, with over 500 cases published in the literature. RCVS presents with recurrent severe thunderclap headaches, with or without neurological symptoms, and diffuse segmental narrowing of the cerebral arteries. The sequelae of the disease may involve subarachnoid hemorrhage, lobar hemorrhage, and watershed infarcts [44]. The condition is associated with precipitating factors such as postpartum periods, eclampsia, preeclampsia, select drugs, medications, and blood products [44]. Exposure to vasoactive substances such as cannabis, serotonin reuptake inhibitors, and cocaine have been implicated in patients with RCVS, with exposure documented in up to 60% of non-postpartum-related RCVS cases [44]. The narrowing of the arteries in RCVS, by definition, is reversible within 3 months [41]. Consequently, scores have been developed to help differentiate RCVS from PACNS, such as that by Rocha and colleagues [30]. They report a 99% specificity and 90% sensitivity with a score ≥ 5 and a 100% specificity and 85% sensitivity for excluding RCVS with a score ≤ 2. Numerous imaging findings are also considered in this score, including the presence of infarcts, parenchymal hemorrhage, and specific vessels affected [30]. 

RCVS is crucial to differentiate from vasculitis because standard treatment of vasculitis, including corticosteroids, worsens RCVS. Moreover, neuroimaging tools have been suggested to provide important differentiating findings to help us make this distinction. Notably, black blood MRI in PACNS can visualize inflammatory changes in multiple vessels characterized by long and smooth circumferential concentric wall thickening with gadolinium enhancement. In contrast, angiograms in RCVS patients usually show short stenosis without enhancement or with moderate wall thickening [45]. Such findings can be compared to those amongst cases with vasculitis to further reveal a pattern through a machine learning algorithm to help physicians better distinguish conditions such as those discussed.

### 4.4. An Approach to Diagnosis

A gestalt approach to the diagnosis of vasculitis, which includes both imaging and clinical presentation, may be critical given that imaging features alone are often inconsistent, ambiguous, or non-detectable. In fact, in 38.6% of histologically confirmed cases of vasculitis, cerebral angiography was normal. In only 25% of the confirmed cases of PACNS, the classic imaging findings of vasculitis were seen. Moreover, diagnostic biopsies also present flaws. Up to 15% of cases do not show the characteristic granulomatous pathology, and sampling error is possible as vasculitis may be a focal condition. Although considered the gold standard, the sensitivity for biopsy has been quoted as only 74.4% [36]. 

Ultimately, most of the literature suggests a diagnosis built upon both clinical suspicion and imaging findings [36]. Findings of clinical symptoms that are relatively common in vasculitis that should raise suspicion and may warrant further investigation include headaches, neurological deficits, and cognitive dysfunction. Given the vague clinical presentation, a combination with imaging such as MRI, which boasts high sensitivity, can be postulated to yield high negative predictive value [36,42]. However, difficulty arises when trying to rule out the condition, given the scarcity of specific findings. For this, AI/ML may play a role. Given the many overlapping signs, a machine learning algorithm may aid in elucidating a complex pattern that would otherwise be difficult to encompass in a singular score. This algorithm may assist in diagnosis by prompting radiologists and clinicians to investigate findings further and through the presentation of a differential diagnosis based on clinical context and imaging findings. 

### 4.5. AI/ML

AI/ML may prove to be of benefit in increasing efficiency in diagnosing vasculitis. In the case that a machine algorithm can yield a high negative predictive value, as has been demonstrated in a study on mammography, this can improve efficiency by reducing the number of normal scans that must be read [46]. Moreover, we suggest feeding a machine learning algorithm multiple levels of information to reveal complex patterns and correlations that are otherwise very time-consuming to interpret or apply [47,48]. One layer of information may derive from the patient’s clinical presentation, such as the patient’s demographics, signs, symptoms, and risk factors (e.g., age, pregnancy, and comorbidities such as hypertension) as this may alter the differential diagnosis [49]. When feeding the algorithm data from many cases, a pattern of clinical presentation may be recognized, allowing for better discrimination of vasculitis conditions versus mimics. This is the strength of machine learning, as it allows for the analysis and identification of patterns based on millions of features that would otherwise be impractically time-consuming to elucidate [48]. In this case, efficiency will be greatly improved as a pattern of symptomology that is quite specific may be revealed, which will help us narrow our differential. Clinical context would be especially crucial in narrowing the differential based on information such as patient age, which can dramatically alter what conditions we consider in the differential. In pediatric and young adult patients, MS is relatively common and a possible mimic of vasculitis and thus must be considered [50]. In contrast, in middle-aged and older patients, RCVS is more common and a mimic of vasculitis [51]. Consideration of demographic information can further guide diagnosis. 

We suggest the next layer of data in this algorithm include imaging findings. The difficulty posed in diagnosing radiological vasculitis lies in the overlapping features and non-specific findings [52]. Radiologists interpreting vasculitis findings and feeding them to this algorithm may allow a constellation of findings to be revealed. An amalgamation of specific radiological findings may thus be identified that indicates a presentation keeping with a likely vasculitis case as opposed to a mimic. After the radiologist interprets the imaging findings and feeds them to the machine, these may be combined with the clinical data entered to formulate a list of differentials and their respective likelihoods. This may aid radiologists and clinicians in making the diagnosis by drawing their attention to important findings and patterns that the algorithm revealed. In theory, AI/ML may augment the process to the point that patients will not need to be subjected to numerous laborious investigations, and clinicians will not be tasked with the complexity of identifying this specific condition in a sea of non-specific findings. 

As we have reviewed (Table 1), a plethora of criteria exist to aid in the diagnosis of the vast spectrum of vasculitis and mimics. However, given the non-specific findings and similarities amongst conditions, a one “catch-all” score is difficult to formulate. Rather, the answer to aid diagnostic efficiency may lie in complex patterns that can be revealed and applied through AI/ML to streamline the diagnosis process [53,54]. An AI/ML algorithm may be able to combine parts of these existing scores to reveal and compute complex patterns of findings to stratify which conditions are most in keeping with the presentation and, in turn, assist the clinicians in making the final diagnosis. Diagnostic ambiguity exists because of how these conditions tend to present so similarly; however, it is equally important for the machine learning algorithm to be able to accurately diagnose mimics of vasculitis. This review outlines the existing neuroimaging scoring criteria in the literature for both vasculitis and common mimics in hopes of providing a strong basis from which to build or further augment an AI/ML algorithm.

For many vasculitis conditions, creating a practical radiological score to differentiate them from mimics will be difficult given the non-specific findings, and a complex score will be inefficient and difficult to implement in practice. Machine learning can help address this by identifying patterns that are too complicated to deduce to a score and then presenting these data to clinicians to make the diagnosis by providing them with impressions based on the patterns revealed through the algorithm. 

An AI/ML algorithm to differentiate MS from mimics has been recently described by Rocca et al. [55]. They tested the algorithm’s ability to diagnose cases of MS, migraines, Neuromyelitis optica spectrum disorder (NOMSD), and CNS vasculitis and reported success. Their algorithm used a neural network, as described by the authors, to create the deep learning algorithm [55]. Their success indicates that the role of AI/ML in augmenting diagnostic capabilities is possible. This strengthens the need for a scoping review such as this to collect and disseminate existing criteria to serve as a foundational report to guide future algorithms. We hope that our review can augment the system developed by Rocca et al. or other similar machine-learning algorithms to expand their capabilities to include other important mimics. Ultimately, our results can serve as foundational information for future AI/ML research in the field of CNS vasculitis diagnosis. 

It is important to note, however, that AI/ML is not realistically viable for end-to-end automation in such conditions [56]. Some standalone automatic diagnostic algorithms have been shown to be inferior to human doctors in diagnostic accuracy and raise the suggestion that perhaps these algorithms would be best suited to augment physicians [57]. Numerous clinical (e.g., evaluation of neurological symptoms) and radiological interpretations (e.g., dissemination in space) afford a level of subjectivity that is determined by experts in the field. Instead, we propose the utilization of experts in the field to make these interpretations and then inputting these findings into an algorithm that can streamline the diagnostic process by acting similarly to a computerized clinical decision support system to increase efficiency [58].

### 4.6. Limitations

While all efforts were made to include relevant articles in the field, it must be acknowledged that the possibility of missed literature exists. Given that our search was limited to two databases (Medline and EMBASE), the literature exclusively found in other databases would be missed. Moreover, our inclusion criteria were limited to work published in the English language, which may narrow our results by excluding relevant work published in other languages. Grey literature that is unpublished in these search engines is also likely to be missed, and thus, the possibility of the relevant literature from this domain being excluded must also be acknowledged. 

## 5. Conclusions

Given the difficulty in diagnosing vasculitis and the lack of single investigations with adequate sensitivity and specificity, a gestalt approach to clinical signs and radiological findings is necessary. Within the literature exists many scores that aim to elucidate and better differentiate vasculitis conditions from common mimics. However, no single score has been effective enough to differentiate these conditions reliably. This review presents the existing scores to serve as a foundational building block for future work aimed at consolidating the existing criteria/scores to develop a pattern to aid in diagnosing these conditions. In conjunction with clinicians and radiologists, we suggest that AI/ML can provide benefits in augmenting our diagnostic ability and efficiency by generating an algorithm to classify the likelihood of vasculitis conditions from common mimics. 

## Figures and Tables

**Table 1:** Current criteria to evaluate CNS vasculitis conditions and common mimics.

Disease	Criteria Name	Year Published	Population	Criteria Variable	Assessment
Multiple Sclerosis	McDonald [15]	2010, revised in 2017	Adults	Imaging, clinical	The McDonald criteria considers dissemination in time and dissemination in space (DIS). Dissemination in space can be demonstrated by one or more T2-hyperintense lesions present in two out of four following areas: Cortical or juxtacortical; Periventricular; Infratentorial; Spinal cord.
	MAGNIMS Criteria [17]	2016	Adults	Imaging	DIS can be demonstrated by the involvement of at least two out of five areas of the CNS as follows: ≥3 periventricular lesions; ≥1 infratentorial lesion; ≥1 spinal cord lesion; ≥1 optic nerve lesion; ≥1 cortical/juxtacortical lesion.
	IPMS Criteria [18]	2007	Pediatric and Adult	Imaging, clinical	Two or more discrete demyelination episodes separated by time and space; In children, the demyelination episodes must not meet the criteria for ADEM.
	Barkhof Criteria [19]	1997	Adults	Imaging	At least one gadolinium-enhancing lesion or at least nine lesions on T2-weighted; At least three periventricular lesions; At least one juxtacortical lesion; At least one infratentorial lesion.
	Paty’s Criteria [20]	1988	Pediatric and Adults	Imaging	Paty’s criteria Presence of four AIS greater than 3 mm; Adults: the presence of three AIS, one periventricular, greater than 3 mm.
PACNS	Calabrese and Mallek Criteria [21]	1988	Adults	Imaging, clinical	The presence of an acquired and otherwise unexplained neurologic deficit With the presence of either classic angiographic or histopathologic features of angiitis within the CNS; No evidence of systemic vasculitis or any condition that could elicit the angiographic or pathologic features.
	Salvarani et al., [22]	2007	Adults	Imaging, clinical	Recent history or presence of an acquired neurological deficit unexplained by other causes; Evidence of vasculitis in a CNS biopsy specimen; A cerebral angiogram with changes characteristic of vasculitis; Exclusion of diseases that might mimic PCNSV, including hypercoagulability, varicella zoster, other infectious vasculitides, and other processes.
	Moore et al. [23]	1989	Adults	Imaging, clinical	Clinical pattern of headaches and multifocal neurologic deficits present for at least 6 months unless the deficits are severe at onset or rapidly progressive; Cerebral angiography demonstrating segmental arterial narrowing; Exclusion of systemic inflammation or infection; Leptomeningeal/parenchymal biopsy demonstrating vascular inflammation or exclusion of alternate diagnoses.
	Alrawi et al. [24]	1999	Adults	Tissue	Minimum of two layers of lymphocytes within or around the walls of parenchymal or leptomeningeal and dural vessels (“lymphocytic inflammation”); Structural alterations of the vessel wall, such as prominence of the endothelial cells, indistinct appearance with or without necrosis; Pink neuronal cytoplasm and pyknotic neuronal nuclei with or without pyknotic glial nuclei and astrocytic gliosis (“ischemic changes”); Neuronophagia; Parenchymal (including perivascular) edema; Exclusion of alternative diagnoses.
	Birnbaum et al. [25]	2009	Adults	Imaging, clinical	Patients receive a definite diagnosis of PACNS if there is confirmation of vasculitis on analysis of a tissue biopsy specimen; Patients have a probable diagnosis of PACNS in the absence of tissue confirmation if there are high-probability findings on an angiogram with abnormal findings on MRI and a CSF profile consistent with PACNS.
cPACNS	Besneler et al. [26]	2006	Pediatric	Imaging, clinical	Newly acquired, otherwise unexplained focal or diffuse neurologic deficits or psychiatric symptoms in a patient less than 18 years of age; Angiographic and/or histopathologic features of central nervous system angiitis; Absence of an underlying or associated systemic disease.
Neuromyelitis Optica (NMO)	Wingerchuk’s Criteria (AQP4 Ab-negative NMO) [27]	2006	Adults	Imaging	Optic neuritis, myelitis. At least two of three supportive criteria: Contiguous spinal cord MRI lesion extending over greater than or equal to three vertebral segments; Brain MRI does not meet diagnostic criteria for multiple sclerosis; NMO-IgG seropositive status.
	Wingerchuk et al. Revised Criteria [28]	2014	Adults	Imaging, clinical	NMOSD with AQP4-IgG At least one core clinical characteristic; Positive test for AQP4-IgG using best available detection method (cell-based assay strongly recommended); Exclusion of alternative diagnoses. NMOSD without AQP4-IgG or MOSD with unknown AQP4-IgG status At least two core clinical characteristics occurring as a result of one or more clinical attacks and meeting all of the following requirements: At least one core clinical characteristic must be optic neuritis, acute myelitis with LETM, or area postrema syndrome; Dissemination in space (two or more different core clinical characteristics); Fulfillment of additional MRI requirements, as applicable. Negative tests for AQP4-IgG using the best available detection methods or testing unavailable; Exclusion of alternative diagnoses. Core Clinical Characteristics Optic neuritis; Acute myelitis; Area postrema syndrome episode of otherwise unexplained hiccups or nausea and vomiting; Acute brainstem syndrome; Symptomatic narcolepsy or acute diencephalic clinical syndrome with NMOSD-typical diencephalic MRI lesions; Symptomatic cerebral syndrome with NMOSD-typical brain lesions. Additional MRI requirements for NMOSD without AQP4-IgG and NMOSD with unknown ACP4-IgG status Acute optic neuritis: required brain MRI showing normal findings or only non-specific white matter lesions, OR optic nerve MRI with T2-hyperintense lesion or T1-weighted gadolinium-enhancing lesion extending over ½ optic nerve length or involving optic chiasm; Acute myelitis: requires associated intramedullary MRI lesion extending over ≥3 contiguous segments (LETM) OR ≥ 3 continuous segments of focal spinal cord atrophy in patients with history compatible with acute myelitis; Area postrema syndrome: requires associated dorsal medulla/area postrema lesions; Acute brainstem syndrome: requires associated periependymal brainstem lesions.
Reversible Cerebral Vasoconstriction Syndromes (RCVS)	Calabrese et al. Criteria [29]	2007	Adults	Imaging, clinical	Transfemoral angiography or indirect CTA or MRA documenting multifocal segmental cerebral artery vasoconstriction; No evidence for aneurysmal subarachnoid hemorrhage; Reversibility of angiographic abnormalities within 12 weeks after onset.
	Rocha et al. RCVS2 criteria [30]	2019	Adults	Imaging, clinical	Criteria (points) Recurrent or single thunderclap headache ⚬ Present (5); ⚬ Absent (0). Carotid artery ⚬ Affected (−2); ⚬ Not affected (0). Vasoconstrictive Trigger ⚬ Present (3); ⚬ Absent (0). Sex ⚬ Female (1); ⚬ Male (0). Subarachnoid hemorrhage ⚬ Present (1); ⚬ Absent (0). Score ≥ 5, high sensitivity and specificity for RCVS diagnosis Score ≤ 2 high sensitivity and specificity for excluding RCVS
Rasmussen Encephalitis	Bien et al. [31]	2005	Adults	Imaging, clinical	Part A: ⚬ Focal seizures (with or without Epilepsia partialis continua) and unilateral cortical deficit(s); ⚬ Unihemispheric slowing with or without epileptiform activity and unilateral seizure onset; ⚬ Unihemispheric focal cortical atrophy and at least one of the following: ⚬ Grey or white matter T2/FLAIR hyperintense signal; ⚬ Hyperintense signal or atrophy of the ipsilateral caudate head. Part B: ⚬ Epilepsia partialis continua or progressive unilateral cortical deficit(s); ⚬ Progressive unihemispheric focal cortical atrophy; ⚬ T cell dominated encephalitis with activated microglial cells (typically, but not necessarily forming nodules) and reactive astrogliosis; ⚬ Numerous parenchymal macrophages, B cells, plasma cells, or viral inclusion bodies exclude the diagnosis of RE.
Myelin oligodendrocyte glycoprotein antibody-associated disease (MOGAD)	2023	Banwell et al. [32]	Adults	Imaging, clinical	The presence of a core clinical demyelinating event ⚬ (e.g., optic neuritis, transverse myelitis, acute disseminated encephalomyelitis (ADEM), cerebral monofcoal or polyfocal deficits, brainstem or cerebellar deficits, and cerebral cortical encephalitis). A positive myelin oligodendrocyte glycoprotein immunoglobulin G (MOG-IgG) antibody test; In cases where serum MOG-IgG titer is low positive, positive without a reported tire, or seronegative, but with clear positivity in cerebrospinal fluid, at least one additional supportive criteria or MRI feature is required along with seronegative aquaporin 4 (AQP4)-IgG; e.g., central cord lesion or axial H-sign on imaging, conus lesion, multiple ill-defined T2-hyperintense lesions in supratentorial and often infratentorial white matter, deep gray matter involvement, ill-defined T2-hyperintensity involving pons, middle cerebellar peduncle, or medulla, cortical lesion with or without lesional and overlying meningeal enhancement.
	2018	Jarius et al. [33]	Adults	Imaging, clinical	MOGAD should be diagnosed in all patients who meet all of the following criteria: Monophasic or relapsing acute ON, myelitis, brainstem encephalitis, or encephalitis, or any combination of these syndromes; MRI or electrophysiological (visual evoked potentials in patients with isolated ON) findings compatible with CNS demyelination; Seropositivity for MOG-IgG as detected by means of a cell-based assay employing full-length human MOG as the target antigen.
	2018	López-Chiriboga et al. [34]	Adults	Imaging, clinical	Laboratory finding: serum positive for MOG-IgG by cell-based assay; Clinical findings: any of the following presentations: ⚬ ADEM Optic neuritis, including CRION Transverse myelitis, brain or brainstem syndrome compatible with demyelination, or any combination of the above. Exclusion of alternative diagnosis.
Primary CNS Vasculitis	2019	Rice and Scolding [35]	Adults	Imaging, clinical	Proposed criteria for the diagnosis of central nervous system (CNS) vasculitis: Definite ⚬ Clinical presentation suggesting CNS vasculitis with the exclusion of alternative possible diagnoses and primary systemic vasculitic syndrome; ⚬ Plus, the presence of positive CNS histology, that is, biopsy or autopsy showing CNS angiitis (granulomatous, lymphocytic, or necrotizing), including evidence of vessel wall damage. Possible ⚬ Clinical presentation compatible with CNS vasculitis with the exclusion of alternative possible diagnoses and primary systemic vasculitic syndrome; ⚬ Plus, laboratory and imaging support for CNS inflammation (elevated levels of cerebrospinal fluid protein and/or cells, and/or the presence of oligoclonal bands and/or MR scan evidence compatible with CNS vasculitis), with angiographic exclusion of other specific entities; ⚬ But without histological proof of vasculitis.

## Data Availability

Data sharing is not applicable.

## Abbreviations

**Abbreviation:**	**Definition:**
AI/ML	Artificial intelligence/machine learning
AIS	Area of increased signal
AQP4-IgG	Anti-aquaporin-4 immunoglobulin G
CA	Conventional angiography
CNS	Central nervous system
CSF	Cerebrospinal fluid
cPACNS	Childhood primary angiitis of the central nervous system
DIS	Dissemination in space
HRVWI	High-resolution vessel wall imaging
IgG	Immunoglobulin G
LETM	Longitudinally extensive transverse myelitis
MRA	Magnetic resonance angiography
MRI	Magnetic resonance imaging
MS	Multiple sclerosis
NMO	Neuromyelitis optica
NMOSD	Neuromyelitis optica spectrum disorder
PACNS	Primary angiitis of the central nervous system
PCNSV	Primary central nervous system vasculitis
RCT	Randomized control trial
RCVS	Reversible cerebral vasoconstriction syndromes
SVV	Small-vessel vasculitis

## Appendix A

### Appendix A.1. Search String

(Inflammatory disease OR Demyelinating Disease OR Acute disseminated encephalomyelitis OR Demyelination OR Leukodystrophy OR Multiple Sclerosis OR Progressive multifocal leukoencephalopathy OR Schilder disease OR Subacute sclerosing panencephalitis OR Central nervous system viral disease OR Encephalitis OR Myelitis OR Autoimmune disease of the nervous system OR Demyelinating autoimmune diseases of the central nervous system OR Rasmussen syndrome OR Stroke OR Central nervous system vasculitis) AND (diagnostic imaging OR CA OR Calcium imaging OR MRI OR Magnetic resonance imaging OR MRA OR magnetic resonance angiogram OR MR angiography) AND (scoring tool OR quantitative measure OR quantitative measurement OR score OR scoring method OR consensus score OR measurement technique OR measuring disease OR scoring mechanism) AND (diagnostic tool OR differential diagnosis tool OR differential diagnosis score OR differential diagnosis mechanism OR diagnostic method)

Limits

- English language
- Remove duplicates
- Year ≥ 2000

### Appendix A.2. Mallek and Calabrese Criteria

They propose the following criteria:

1. The presence of a newly acquired, unexplained neurologic deficit after thorough clinical and laboratory evaluation;
2. Evidence of vasculitis within the central nervous system on cerebral angiography and/or brain biopsy;
3. No evidence of systemic vasculitis or any other condition to which the angiographic or pathologic features could be secondary or that could mimic the process.

## References

[1] Handel A.E., Giovannoni G., Ebers G.C., Ramagopalan S.V. (2010). Environmental Factors and Their Timing in Adult-Onset Multiple Sclerosis. Nat. Rev. Neurol..

[2] Dobson R., Giovannoni G. (2019). Multiple Sclerosis—A Review. Eur. J. Neurol..

[3] Brownlee W.J., Hardy T.A., Fazekas F., Miller D.H. (2017). Diagnosis of Multiple Sclerosis: Progress and Challenges. Lancet.

[4] Fisniku L.K., Brex P.A., Altmann D.R., Miszkiel K.A., Benton C.E., Lanyon R., Thompson A.J., Miller D.H. (2008). Disability and T2 MRI Lesions: A 20-Year Follow-up of Patients with Relapse Onset of Multiple Sclerosis. Brain.

[5] Twilt M., Benseler S.M. (2012). The Spectrum of CNS Vasculitis in Children and Adults. Nat. Rev. Rheumatol..

[6] Hutchinson C., Elbers J., Halliday W., Branson H., Laughlin S., Armstrong D., Hawkins C., Westmacott R., Benseler S.M. (2010). Treatment of Small Vessel Primary CNS Vasculitis in Children: An Open-Label Cohort Study. Lancet Neurol..

[7] Abdel Razek A.A.K., Alvarez H., Bagg S., Refaat S., Castillo M. (2014). Imaging Spectrum of CNS Vasculitis. RadioGraphics.

[8] Benseler S.M., deVeber G., Hawkins C., Schneider R., Tyrrell P.N., Aviv R.I., Armstrong D., Laxer R.M., Silverman E.D. (2005). Angiography-Negative Primary Central Nervous System Vasculitis in Children: A Newly Recognized Inflammatory Central Nervous System Disease. Arthritis Rheum..

[9] Ömerhoca S., Akkaş S.Y., İçen N.K. (2018). Multiple Sclerosis: Diagnosis and Differential Diagnosis. Noro Psikiyatr. Ars..

[10] Eiden S., Beck C., Venhoff N., Elsheikh S., Ihorst G., Urbach H., Meckel S. (2019). High-Resolution Contrast-Enhanced Vessel Wall Imaging in Patients with Suspected Cerebral Vasculitis: Prospective Comparison of Whole-Brain 3D T1 SPACE versus 2D T1 Black Blood MRI at 3 Tesla. PLoS ONE.

[11] Schuster S., Bachmann H., Thom V., Kaufmann-Buehler A.-K., Matschke J., Siemonsen S., Glatzel M., Fiehler J., Gerloff C., Magnus T. (2017). Subtypes of Primary Angiitis of the CNS Identified by MRI Patterns Reflect the Size of Affected Vessels. J. Neurol. Neurosurg. Psychiatry.

[12] Alexander M.D., Yuan C., Rutman A., Tirschwell D.L., Palagallo G., Gandhi D., Sekhar L.N., Mossa-Basha M. (2016). High-Resolution Intracranial Vessel Wall Imaging: Imaging beyond the Lumen. J. Neurol. Neurosurg. Psychiatry.

[13] Salvarani C., Brown R.D.J., Christianson T., Miller D.V., Giannini C., Huston J.I., Hunder G.G. (2015). An Update of the Mayo Clinic Cohort of Patients with Adult Primary Central Nervous System Vasculitis: Description of 163 Patients. Medicine.

[14] von Elm E., Altman D.G., Egger M., Pocock S.J., Gøtzsche P.C., Vandenbroucke J.P. (2007). The Strengthening the Reporting of Observational Studies in Epidemiology (STROBE) Statement: Guidelines for Reporting Observational Studies. Ann. Intern. Med..

[15] Thompson A.J., Banwell B.L., Barkhof F., Carroll W.M., Coetzee T., Comi G., Correale J., Fazekas F., Filippi M., Freedman M.S. (2018). Diagnosis of Multiple Sclerosis: 2017 Revisions of the McDonald Criteria. Lancet Neurol..

[16] Pereira F.V., de Menezes Jarry V., Castro J.T.S., Appenzeller S., Reis F. (2021). Pediatric Inflammatory Demyelinating Disorders and Mimickers: How to Differentiate with MRI?. Autoimmun. Rev..

[17] Filippi M., Rocca M.A., Ciccarelli O., De Stefano N., Evangelou N., Kappos L., Rovira A., Sastre-Garriga J., Tintorè M., Frederiksen J.L. (2016). MRI Criteria for the Diagnosis of Multiple Sclerosis: MAGNIMS Consensus Guidelines. Lancet Neurol..

[18] Krupp L.B., Banwell B., Tenembaum S. (2007). Consensus Definitions Proposed for Pediatric Multiple Sclerosis and Related Disorders. Neurology.

[19] Barkhof F., Filippi M., Miller D.H., Scheltens P., Campi A., Polman C.H., Comi G., Adèr H.J., Losseff N., Valk J. (1997). Comparison of MRI Criteria at First Presentation to Predict Conversion to Clinically Definite Multiple Sclerosis. Brain.

[20] Paty D.W., Oger J.J.F., Kastrukoff L.F., Hashimoto S.A., Hooge J.P., Eisen A.A., Eisen K.A., Purves S.J., Low M.D., Brandejs V. (1988). MRI in the Diagnosis of MS. Neurology.

[21] Calabrese L., Mallek J. (1988). Primary Angiitis of the Central Nervous System. Report of 8 New Cases, Review of the Literature, and Proposal for Diagnostic Criteria. Medicine.

[22] Salvarani C., Brown R.D., Calamia K.T., Christianson T.J.H., Weigand S.D., Miller D.V., Giannini C., Meschia J.F., Huston III J., Hunder G.G. (2007). Primary Central Nervous System Vasculitis: Analysis of 101 Patients. Ann. Neurol..

[23] Moore P.M. (1989). Diagnosis and Management of Isolated Angiitis of the Central Nervous System. Neurology.

[24] Alrawi A., Trobe J.D., Blaivas M., Musch D.C. (1999). Brain Biopsy in Primary Angiitis of the Central Nervous System. Neurology.

[25] Birnbaum J., Hellmann D.B. (2009). Primary Angiitis of the Central Nervous System. Arch. Neurol..

[26] Benseler S.M., Silverman E., Aviv R.I., Schneider R., Armstrong D., Tyrrell P.N., deVeber G. (2006). Primary Central Nervous System Vasculitis in Children. Arthritis Rheum..

[27] Wingerchuk D.M., Lennon V.A., Pittock S.J., Lucchinetti C.F., Weinshenker B.G. (2006). Revised Diagnostic Criteria for Neuromyelitis Optica. Neurology.

[28] Wingerchuk D., Banwell B., Bennett J., Cabre P., Carroll W., Chitnis T., De Seze J., Fujihara K., Greenberg B., Jacob A. (2014). Revised Diagnostic Criteria for Neuromyelitis Optica Spectrum Disorders (S63.001). Neurology.

[29] Calabrese L.H., Dodick D.W., Schwedt T.J., Singhal A.B. (2007). Narrative Review: Reversible Cerebral Vasoconstriction Syndromes. Ann. Intern. Med..

[30] Rocha E.A., Topcuoglu M.A., Silva G.S., Singhal A.B. (2019). RCVS_2_ Score and Diagnostic Approach for Reversible Cerebral Vasoconstriction Syndrome. Neurology.

[31] Bien C.G., Granata T., Antozzi C., Cross J.H., Dulac O., Kurthen M., Lassmann H., Mantegazza R., Villemure J.-G., Spreafico R. (2005). Pathogenesis, Diagnosis and Treatment of Rasmussen Encephalitis: A European Consensus Statement. Brain.

[32] Banwell B., Bennett J.L., Marignier R., Kim H.J., Brilot F., Flanagan E.P., Ramanathan S., Waters P., Tenembaum S., Graves J.S. (2023). Diagnosis of Myelin Oligodendrocyte Glycoprotein Antibody-Associated Disease: International MOGAD Panel Proposed Criteria. Lancet Neurol..

[33] Jarius S., Paul F., Aktas O., Asgari N., Dale R.C., de Seze J., Franciotta D., Fujihara K., Jacob A., Kim H.J. (2018). MOG Encephalomyelitis: International Recommendations on Diagnosis and Antibody Testing. J. Neuroinflammation.

[34] López-Chiriboga A.S., Majed M., Fryer J., Dubey D., McKeon A., Flanagan E.P., Jitprapaikulsan J., Kothapalli N., Tillema J.-M., Chen J. (2018). Association of MOG-IgG Serostatus With Relapse After Acute Disseminated Encephalomyelitis and Proposed Diagnostic Criteria for MOG-IgG–Associated Disorders. JAMA Neurol..

[35] Rice C.M., Scolding N.J. (2020). The Diagnosis of Primary Central Nervous System Vasculitis. Pract. Neurol..

[36] Calabrese L.H., Furlan A.J., Gragg L.A., Ropos T.J. (1992). Primary Angiitis of the Central Nervous System: Diagnostic Criteria and Clinical Approach. Clevel. Clin. J. Med..

[37] Beuker C., Strunk D., Rawal R., Schmidt-Pogoda A., Werring N., Milles L., Ruck T., Wiendl H., Meuth S., Minnerup H. (2021). Primary Angiitis of the CNS. Neurol. Neuroimmunol. Neuroinflamm..

[38] Hajj-Ali R.A., Singhal A.B., Benseler S., Molloy E., Calabrese L.H. (2011). Primary Angiitis of the CNS. Lancet Neurol..

[39] Hajj-Ali R.A. (2010). Primary Angiitis of the Central Nervous System: Differential Diagnosis and Treatment. Best Pract. Res. Clin. Rheumatol..

[40] Stone J., Pomper M., Roubenoff R., Miller T., Hellmann D. (1994). Sensitivities of Noninvasive Tests for Central Nervous System Vasculitis: A Comparison of Lumbar Puncture, Computed Tomography, and Magnetic Resonance Imaging. J. Rheumatol..

[41] Cappelen-Smith C., Calic Z., Cordato D. (2017). Reversible Cerebral Vasoconstriction Syndrome: Recognition and Treatment. Curr. Treat. Options Neurol..

[42] Deb-Chatterji M., Schuster S., Haeussler V., Gerloff C., Thomalla G., Magnus T. (2019). Primary Angiitis of the Central Nervous System: New Potential Imaging Techniques and Biomarkers in Blood and Cerebrospinal Fluid. Front. Neurol..

[43] Sundaram S., Kumar P.N., Sharma D.P., Kesavadas C., Sreedharan S.E., Prasad B.A., Sylaja P. (2021). High-Resolution Vessel Wall Imaging in Primary Angiitis of Central Nervous System. Ann. Indian Acad. Neurol..

[44] Ducros A., Boukobza M., Porcher R., Sarov M., Valade D., Bousser M.-G. (2007). The Clinical and Radiological Spectrum of Reversible Cerebral Vasoconstriction Syndrome. A Prospective Series of 67 Patients. Brain.

[45] Beuker C., Schmidt A., Strunk D., Sporns P.B., Wiendl H., Meuth S.G., Minnerup J. (2018). Primary Angiitis of the Central Nervous System: Diagnosis and Treatment. Ther. Adv. Neurol. Disord..

[46] Kyono T., Gilbert F.J., van der Schaar M. (2020). Improving Workflow Efficiency for Mammography Using Machine Learning. J. Am. Coll. Radiol..

[47] Wang S., Summers R.M. (2012). Machine Learning and Radiology. Med. Image Anal..

[48] Rajkomar A., Dean J., Kohane I. (2019). Machine Learning in Medicine. N. Engl. J. Med..

[49] Cannistraro R.J., Badi M., Eidelman B.H., Dickson D.W., Middlebrooks E.H., Meschia J.F. (2019). Meschia CNS Small Vessel Disease. Neurology.

[50] Jeong A., Oleske D.M., Holman J. (2019). Epidemiology of Pediatric-Onset Multiple Sclerosis: A Systematic Review of the Literature. J. Child Neurol..

[51] Sattar A., Manousakis G., Jensen M.B. (2010). Systematic Review of Reversible Cerebral Vasoconstriction Syndrome. Expert Rev. Cardiovasc. Ther..

[52] Krawczyk M., Barra L.J., Sposato L.A., Mandzia J.L. (2021). Primary CNS Vasculitis: A Systematic Review on Clinical Characteristics Associated with Abnormal Biopsy and Angiography. Autoimmun. Rev..

[53] Tran J., Sharma D., Gotlieb N., Xu W., Bhat M. (2022). Application of Machine Learning in Liver Transplantation: A Review. Hepatol. Int..

[54] Choy G., Khalilzadeh O., Michalski M., Do S., Samir A.E., Pianykh O.S., Geis J.R., Pandharipande P.V., Brink J.A., Dreyer K.J. (2018). Current Applications and Future Impact of Machine Learning in Radiology. Radiology.

[55] Rocca M.A., Anzalone N., Storelli L., Del Poggio A., Cacciaguerra L., Manfredi A.A., Meani A., Filippi M. (2021). Deep Learning on Conventional Magnetic Resonance Imaging Improves the Diagnosis of Multiple Sclerosis Mimics. Investig. Radiol..

[56] Richens J.G., Lee C.M., Johri S. (2020). Improving the Accuracy of Medical Diagnosis with Causal Machine Learning. Nat. Commun..

[57] Semigran H.L., Levine D.M., Nundy S., Mehrotra A. (2016). Comparison of Physician and Computer Diagnostic Accuracy. JAMA Intern. Med..

[58] Sutton R.T., Pincock D., Baumgart D.C., Sadowski D.C., Fedorak R.N., Kroeker K.I. (2020). An Overview of Clinical Decision Support Systems: Benefits, Risks, and Strategies for Success. NPJ Digit. Med..

